# Evaluation of Design Procedure and Performance of Continuously Reinforced Concrete Pavement According to AASHTO Design Methods

**DOI:** 10.3390/ma15062252

**Published:** 2022-03-18

**Authors:** Milad Moharekpour, Pengfei Liu, Joshua Schmidt, Markus Oeser, Ruxin Jing

**Affiliations:** 1 Institute of Highway Engineering, RWTH Aachen University, D52074 Aachen, Germany; moharekpour@isac.rwth-aachen.de (M.M.); joshua.schmidt@rwth-aachen.de (J.S.); oeser@isac.rwth-aachen.de (M.O.); 2 Pavement Engineering, Delft University of Technology, 2628 CN Delft, The Netherlands

**Keywords:** CRCP, concrete pavement, crack width, crack spacing, AASHTO, MEPDG

## Abstract

The Guide for Design of Pavement Structures (AASHTO 86/93) and Mechanistic Empirical Pavement Design Guide (MEPDG) are two common methods to design continuously reinforced concrete pavement (CRCP) published by the American Association of State Highway and Transportation Officials (AASHTO) in the USA. The AASHTO 86/93 is based on empirical equations to assess the performance of highway pavements under moving loads with known magnitude and frequency derived from experiments on AASHTO road tests. The MEPDG is a pavement design method based on engineering mechanics and numerical models for analysis. It functions by incorporating additional attributes such as environment, material properties, and vehicle axle load to predict pavement performance and degradation at the selected reliability level over the intended performance period. In order to evaluate the CRCP design procedure and performance, crack width (CW) and crack spacing (CS) from five examined test tracks in Europe with different climate condition, base layer, geometry, and materials were collected in this paper and compared with predicted distresses as well as CW and CS from AASHTO 86/93 and MEPDG design methods. The results show that the interactions between geometrics, material properties, traffic, and environmental conditions in the MEPDG method are more pronounced than in the AASHTO 86/93 and the prediction of CS and CW based on MEPDG matched closely with the recorded data from sections.

## 1. Introduction

**Figure 1:**
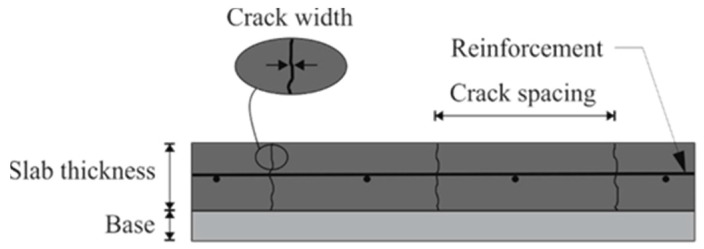
Structure of CRCP and main influencing factors.

Continuously reinforced concrete pavement (CRCP) is a unique rigid pavement with continuous steel bars throughout its length and no contraction transverse joints. It is used mainly in highways and airports to accommodate heavily loaded traffic because of high yield and tensile strengths characteristics [1,2,3,4]. CRCP is constructed with longitudinal reinforcement distributed along the pavement to hold the developing transverse cracks tightly closed, increase load transfer efficiency (LTE) across cracks, and provide stiffness by restraining end movement caused by concrete volumetric changes in the pavement slab under environmental loads [3,5,6]. CRCP is preferred on high-priority routes due to its stability, durability, low maintenance requirements, high availability, and reduction of user delays caused by frequent maintenance and rehabilitation [7]. As CRCP is constructed without any transverse joints, no repair and maintenance of joints are required which causes a significantly lower life cycle cost compared with jointed plain concrete pavement (JPCP) [8]. The performance of CRCP is mainly related to material, construction factors, and crack patterns such as crack spacing (CS), crack width (CW), and steel stress (SS) [5,9,10]. Figure 1 provides the structure of CRCP and the main influencing factors on the behavior of CRCP.

An increase in the amount of longitudinal reinforcement will cause a decrease in the CW in CRCP because of immoderate cracking and loss of integrity of the pavement to act as a continuous slab; the aggregate interlock increases the load transfer, SS decreases, the number of cracks increases, and stiffness at the transverse cracks improves [11,12]. The CW is an important performance factor of CRCP because it controls the aggregate interlock and subsequent shear load transfer capabilities between concrete segments [13] and is affected by concrete placement temperature and season, coarse aggregate type, drying shrinkage, bond-slip relationship between concrete and steel, steel reinforcement, subbase friction, and thermal movement [6]. The CS is a critical factor in the performance of the CRCP. For a well-performing CRCP, the Guide for Design of Pavement Structures (AASHTO 86/93) suggests that the CS should be between 1.07 and 2.44 m (3.5 and 8 ft) [14,15,16]. In CRCP, full-depth punchout, which is determined by the maximum tensile stress at the top of the concrete slab, is the only structural distress and besides the ride in terms of the international roughness index (IRI) are important performance indicators based on the American Association of State Highway and Transportation Officials (AASHTO) Interim Mechanistic Empirical Pavement Design Guide (MEPDG) [17,18]. The aim of the MEPDG is to identify the physical causes of stresses in pavement structures (mechanistic) and calibrate them with observed pavement performance (empirical) [19]. Larger spaced cracks generally could lead to horizontal cracking and cause larger CWs, which can lead to more spalling and more intrusion of incompressible substances and shorter CS increase in the potential of punchouts once the load transfer and support condition deteriorate [20,21]. Smaller CWs increase the ability of the crack to transfer repeated shear stresses (resulting from heavy axle loads) between adjacent slab segments over the long term. Wider cracks exhibit progressively lower LTE over time and traffic, which results in increased load-related critical tensile stresses at the top of the slab, followed by increased fatigue damage and punchouts. The new CRCP design procedure described in the MEPDG, which is currently a paving/industry standard, recommends a CS of 0.91 to 1.83 m (3 to 6 ft) [15,22,23,24,25]. CW affects the performance and service life of CRCP. A maximum allowable CW of 1.0 mm (0.04 in) at the pavement surface was recommended in the AASHTO 86/93 guide based on the considerations of spalling and water penetration. However, CWs of 0.6 mm (0.024 in) or less have also been shown to be more effective in reducing water penetration, thus minimizing corrosion of the steel and maintaining the stability of the base layers and ensuring high LTE (greater than 80 to 90 percent) and decrease the maximum tensile stress in the concrete slab [18,26]. The AASHTO Interim MEPDG forecasts and recommends a maximum CW of 0.5 mm (0.020 in) at steel depth over the entire design life to maintain crack LTE at high levels and minimize possible reinforcement corrosion [24].

European countries such as Belgium, France, and the United Kingdom have a long history with CRCP since 1946, with Belgium being the first country in Europe to trial the CRCP construction methods. In Germany, CRCP is still in the trial phase. From 1977, the following five trial and test sections with a total length of 7700 m were carried out on motorways, federal roads, and a private road: A5 near Darmstadt, A94 near Forstinning, A5 near Bruchsal, private road near Geseke, and B56 near Dueren. In the Belgium route network more than 18 million m^2^ of CRCP were placed [27]. In Poland, the construction of concrete pavements was not considered until the beginning of the 1990s due to energy crises and the increase of the vehicle axle loads to 11.5 tons as well as the increase in traffic intensity [28]. Each individual test track has a specific task and was accompanied by scientists and measurements technologies. 

**Figure 2:**
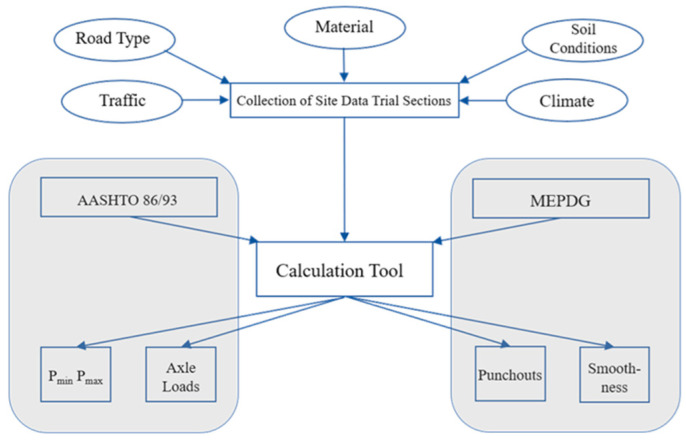
Methodology to compare AASHTO vs. examined tracks.

The objective of this paper (Figure 2) is to closely evaluate and compare the CRCP design procedure and performance based on long-term experiences from Europe with the AASHTO 86/93 and the MEPDG. The individual steps explained in the guides were followed in each case to calculate CW and CS and then compared with the results from the experimental CRCP test sections.

## 2. Selected Test Tracks in This Study

**Figure 3:**
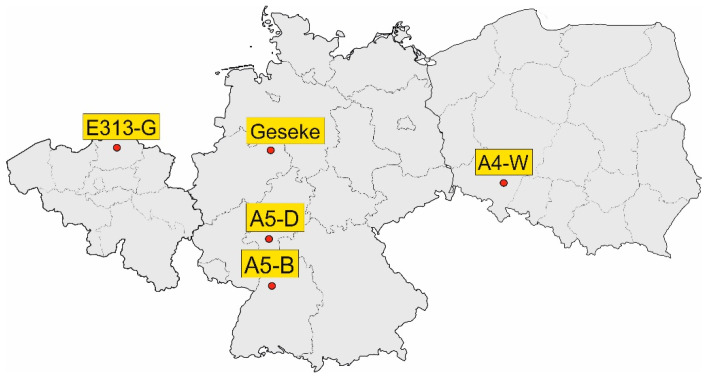
Examined test tracks in CRCP.

In order to compare CRCP design procedures with experience from test tracks, a total of five tracks in Germany, Poland, and Belgium were considered. Figure 3 shows the examined test tracks in Germany, Poland, and Belgium.

**Figure 4:**
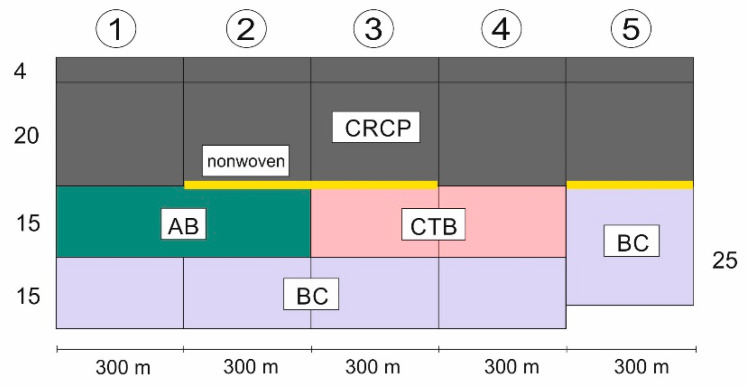
Longitudinal section of motorway A5 Darmstadt. AB: asphalt base layer, CTB: cement bound base layers (hydraulically bound), and BC: stabilization bounded cement.

A 1.52 km long section on the A5 motorway near Darmstadt in Germany was installed in 2004 to investigate five different base layers of CRCP under heavily loaded traffic. The section was divided into five subsections with different base courses, each 300 m long consisting of: (1) asphalt base, (2) asphalt base and nonwoven geotextile, (3) hydraulically bound base, (4) hydraulically bound base and nonwoven geotextile, and (5) stabilization bounded cement. The task of nonwoven geotextile is to prevent additional interlock/bonding between the slab and base, which avoids increasing the effective CRCP slab thickness and reducing the effective steel percentage. The geotextile as a debonding layer is placed between the base layer and the concrete pavement. The placement of a geotextile has the advantage that a drainage can be present, but the risk exists that the concrete flows through the geotextile and makes a stiff bond between the pavement and the base layer. Moreover, it is not proven that the drainage capacity of the geotextile will last in time. By placing an asphalt interlayer, not only will the risk of reflective cracking be diminished, but the behavior of the pavement will alter, as it will act on its own, without jeopardizing the friction with the base layer. The asphalt interlayer will also protect the base layer from erosion. The longitudinal section of motorway A5 Darmstadt is shown in Figure 4.

In 2005, a test section was constructed in a 1.1 km on the A4 motorway in Poland southwest of Breslau with a thickness of 23 cm on a 20 cm thick lean concrete to test CRCP in the field for the first time in Poland. In winter, the temperature often falls below the −15 °C limit. In contrast, in hot summer months, the temperature exceeds 35 °C [29]. The average daily traffic was reported to be 23,785 vehicles in 2015, with a heavy traffic percentage of 24%. 

In 2009, a 1.1 km long CRCP plant road was completed in Geseke in North Rhine-Westphalia (Germany) on a 10 cm thick asphalt base course on the traffic area of Heidelberg Cement AG, which operates a cement plant there and is used by approximately 450 trucks every day. 

The rehabilitation of the E313 at Grobbendonk in Belgium was conducted in 2012. This was one of the first CRCP tracks where the principle of crack initiation was applied. The section on the E313 was constructed according to the current CRCP standard in Belgium: a 25 cm thick CRCP slab with a concrete composition with entrained air, placed on a 5 cm asphalt interlayer and a 25 cm lean concrete base. The longitudinal reinforcement steel amounts to 0.75%, and the position of the longitudinal steel reinforcement is 90 mm (to the center of the bars) below the pavement surface.

**Figure 5:**
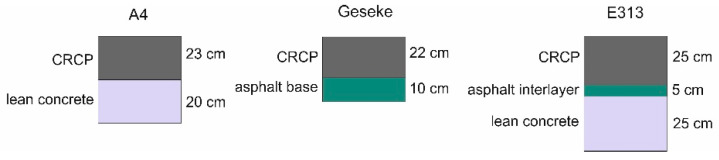
Design structure of test roads: Geseke (**left**), E313 (**middle**), and A4 (**right**).

The design structures of the test roads A4 in Poland, Geseke in Germany, and E313 in Belgium are illustrated in Figure 5.

**Figure 6:**
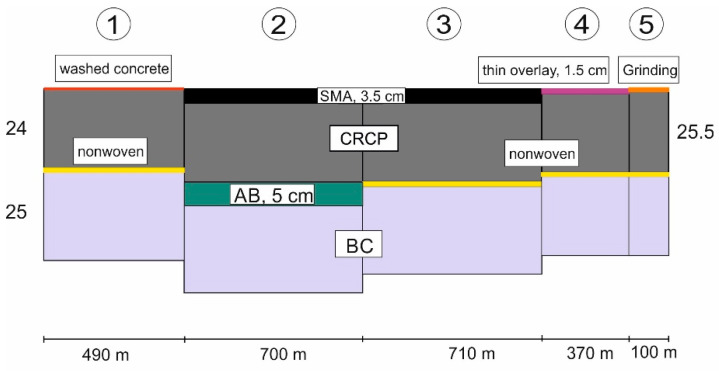
Longitudinal section of motorway A5 near Bruchsal. AB: asphalt base layer, BC: stabilization bounded cement, and SMA: stone mastic asphalt.

The current test track in Germany was constructed in 2015 with a thickness of 24 to 25.5 cm and a length of 2.27 km as a small section of the A5 motorway near Bruchsal north of Karlsruhe. The track is divided into five subsections with different lengths and base layers in order to investigate and optimize skid resistance and noise emission by various surface textures. The ratio of longitudinal reinforcement with 20 mm thick bars was designed to 0.75% (current state 0.61–0.69%). Its longitudinal section is shown in Figure 6.

The non-uniform layers with different structures allow a better comparison in terms of underlayer and interlayer considering the same traffic and climate. These were separately analyzed in the calculations. The technical data of the examined CRCP test tracks are summarized in Table 1.

## 3. Design Standards Used in This Study

Various design methods for determining the slab thickness and the required amount of reinforcement in CRCP have been developed. The Dutch design method in the Netherlands (VENCON 2.0, CROW, Ede, The Netherlands) was developed in 2005. The equations that have been chosen for calculating both traffic and temperature loads are based on the Eisenman [30] and Westergaard [14] methods and relate to the slab thickness and the amount/position of the steel bars. The material stiffness, subgrade moisture, and nonlinear temperature gradient have not been considered. Since 1970, Belgium has developed three design concepts for CRCP. The current concept insists of 25 cm slab thickness on a 5 cm asphalt interlayer and a 150 mm roller compacted concrete base. The longitudinal reinforcement degree is 0.75% and the concrete cover of longitudinal reinforcement is 9 cm [11].

The two primary design methods for CRCP based on their common use and the levels of validation are the AASHTO 86/93 Guide for Design of Pavement Structures and the interim AASHTO MEPDG. The objective of the AASHTO method is the determination of the longitudinal reinforcement ratio that directly affects the CS, CW, and SS [29]. The selection of the reinforcement is carried out based on the desired range of CS, maximum CW, and maximum stress of reinforcement. AASHTO released the MEPDG in 2008, a pavement design methodology based on engineering mechanics. The main difference between the two methods is that AASHTO 86/93 works exclusively empirically based on the results of the AASHTO road test in the late 1950s, while the MEPDG develops a deterministic component for prediction of CRCP performance. The key performance indicators in MEPDG are the accurate prediction of the number of punchouts per mile and roughness, measured with the International Roughness Index (IRI) throughout the design period [31]. In the following, first, these two methods and their influencing factors as well as input parameters are presented, and then a calculation tool for computing these algorithms was developed. With the input of parameters, the frame of the reinforcement ratio for AASHTO 86/93 and punchout prediction and smoothness for MEPDG is calculated.

### 3.1. AASHTO 86/93

**Figure 7:**
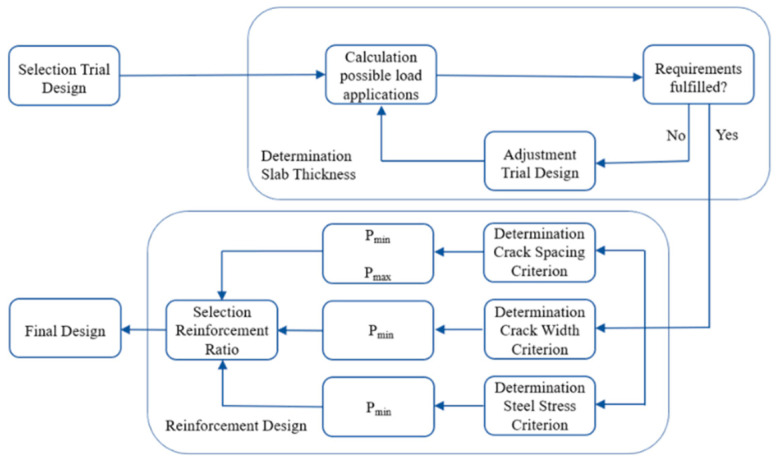
The procedure for dimensioning according to the AASHTO 86/93 method.

The AASHTO design model was developed from data achieved during AASHTO road testing from 1958 to 1960 with further modification based on theory and experience [32]. This design method consists of two calculations of slab thickness and longitudinal reinforcement based on a desired span of CS, maximum CW, and maximum SS [33,34,35], as shown in Figure 7.

The slab thickness design depends on the maximum amount of the 80 kN (18-kip) single axle load over the lifetime of the CRCP to calculate the damage to the pavement caused by the traffic. The resulting thickness design equation depends on a predicted number of the 80 kN (18-kip) single axle load over the design life and is given as follows [14]:

(1) $${\mathrm{Log}}_{10}W_{18}{=Z}_{R}\cdot S_{0}+7.35\cdot {\mathrm{Log}}_{10}\left(D+1\right)-0.06+\frac{{\mathrm{Log}}_{10}\;\left(\frac{\Delta \mathrm{PSI}}{4.5-1.5}\right)}{1+\frac{1.624{\times 10}^{7}}{{\left(D+1\right)}^{8.46}}}+\left(4.22-0.32p_{i}\right)\cdot {\mathrm{Log}}_{10}[\frac{S_{c}^{\prime }\cdot C_{d}\left[D^{0.75}-1.132\right]}{215.63\cdot J\;\left[D^{0.75}-\frac{18.42}{{\left(\frac{E_{c}}{k}\right)}^{0.25}}\right]}]$$

The AASHTO model depends on traffic, pavement structure, and pavement performance and its input requirements include the thickness of CRCP pavement dependent on the reliability (Z_R_), overall standard deviation (S_0_), serviceability (ΔPSI), present serviceability index (PSI), terminal serviceability index (p_t_), PCC modulus of rupture (S_c_), drainage coefficient (c_d_), load transfer coefficient (J), PCC modulus of elastic (E_c_), and effective modulus of subgrade reaction (k), with the slab thickness being the output. The effective modulus of subgrade reaction (k) summarizes all influences that affect the support of the slab from the underground and depends on the resilient modulus of subbase (M_R_), thickness of subbase, loss of Service, depth to bedrock, and elasticity modulus of the subbase. It is therefore necessary to convert M_R_ to k. As with M_R_, the values of k also vary with the season of the year, and the relative damage caused by the change of k needs to be evaluated [30]. For two or more subbase layers it is necessary to determine the composite modulus of subgrade reaction (k_∞_). Then, the relative damage to the CRCP (u_r_) based on slab thickness and the composite k value is determined. Based on the average u_r_ and loss of service, the calculated average value for k must be revised. 

The AASHTO model requires the design of the steel reinforcement to produce desirable crack spacing and crack width and to keep steel stress within allowable levels. To determine the required reinforcement, all three criteria must be verified for the given values. As output, the Equations (2)–(4) calculate three p_min_ and one p_max_ regarding the three criteria entered to satisfy three limiting criterions, CS, CW, and SS. The minimum required amount of steel (P_min_) corresponds to the largest calculated P value from the following equations regarding CS of 2.4 m (8 ft); CW and SS and the maximum required reinforcement (P_max_) correspond to the CS of 1.1 m (3.5 ft).

The formula to calculate the maximum and minimum amount of longitudinal steel relating to the CS is the following [14]:

(2) $$P=\frac{1.062{\left(1+\frac{f_{t}}{1000}\right)}^{1.457}{\left(1+\frac{\alpha_{s}}{2\alpha_{c}}\right)}^{0.25}{(1+\emptyset )}^{0.476}}{{\left(\bar{X}\right)}^{0.217}{(1+\frac{\sigma_{w}}{1000})}^{1.13}{(1+1000Z)}^{0.389}}-1$$

where, (f_t_) is the tensile strength of concrete; (α_s_/α_c_) is the thermal coefficient ratio (steel/concrete); (∅) is the bar diameter; (σw) is the tensile stress due to traffic; and (Z) is the concrete shrinkage at 28 days.

Regarding to spalling and water penetration to avoid corrosion, the CW must be limited and should not exceed 1.0 mm (0.04 in) in the service life. Equation (3) shows the calculation of the steel percentage depending on the defined criterion for the maximum CW [14]:

(3) $$P=\frac{0.358{\left(1+\frac{f_{t}}{1000}\right)}^{1.435}{(1+\emptyset )}^{0.484}}{{\left(\mathrm{CW}\right)}^{0.220}{(1+\frac{\sigma_{w}}{1000})}^{1.079}}-1$$

The predicted CW should be minimized by selecting a higher reinforcement ratio or reducing the bar diameter. AASHTO recommends limiting SS by 75 percent of the ultimate tensile strength of the steel to avoid plastic deformation [11]. For a maximum given SS, the minimum reinforcement ratio can be calculated by the following equation [14]:

(4) $$P=\frac{50.834{\left(1+\frac{\Delta T_{D}}{100}\right)}^{0.135}{(1+\frac{f_{t}}{1000})}^{1.493}}{\sigma_{s}^{0.365}{(1+\frac{\sigma_{w}}{1000})}^{1.146}{\left(1+1000Z\right)}^{0.180}}-1$$

where, (ΔT_D_) is the design temperature drop, which is the difference between the average daily high temperature during the month which the pavement was constructed; (s) is the average daily low temperature during the coldest month of the year; and (σ_s_) is the allowable SS. 

The maximum and minimum number of reinforcing bars is dependent on the calculated p_min_ and p_max,_ the total width of pavement section, (W_S_), the thickness of a slab, D, and the reinforcement bar diameter, Φ, and can be computed by [14]:

(5) $$N_{\min}=0.01273P_{\min}W_{s}D/\emptyset^{2}$$

(6) $$N_{\max}=0.01273P_{\max}W_{s}D/\emptyset^{2}$$

To verify the final reinforcement design, the number of reinforcement bars should be converted into reinforcement grade and calculated backwards by using the equation at the top of each chart to estimate the CS, CW, and SS.

### 3.2. MEPDG

In AASHTO Interim MEPDG design procedure, specific mechanistic-empirical models for the prediction of CRCP performance were developed in 2008, based on engineering mechanics that were nationally calibrated using in-service pavement performance data by the National Cooperative Highway Research Program (NCHRP). A common feature of MEPDG similar to AASHTO 86/93 is that an initial design for the pavement structure is defined at the beginning of the design process, which is then tested for its performance properties and may have to be adjusted after the calculations have been completed. The big difference to AASHTO 1993 is the incremental approach of the design process. All material parameters are defined for the starting time *i*. After running through all equations in the tool, the time increment is set to *i* + 1 and all parameters are recalculated, including material parameters for which a corresponding ageing is considered. The process starts with the selection of a trial design including layer thicknesses, materials, reinforcement, shoulder, and construction information. Site-specific conditions including environment, foundation, and traffic are also considered [11]. 

**Figure 8:**
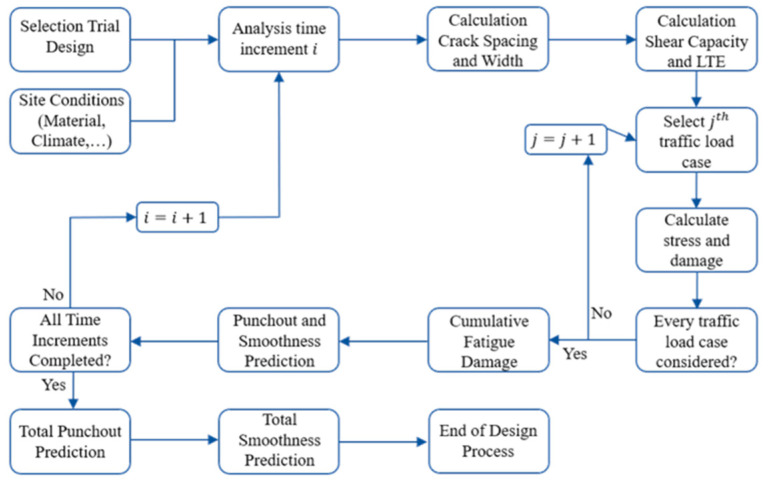
The procedure for dimensioning according to the MEPDG method.

Accordingly, the key to the MEPDG CRCP design procedures is the accurate prediction of the international roughness index (IRI) and punchouts, which are quite complicated to calculate and depend on many parameters [36], as shown in Figure 8.

If the predicted punchouts and IRI at the end of the design period meet the preselected criteria, the design is accepted, otherwise a new design is selected and the pavement system is analyzed [37]. The prediction procedure of the CRCP punchout consists of process input parameters and determines the mean CS, mean CW, LTE, traffic parameters, damage for each design increment, and number of punchouts per mile at the end of each design increment. Equation (7) presents the calculation of the mean CS in the MEPDG:

(7) $$L=\frac{{(f}_{t}-C\times \sigma_{0}\times \left(1-\frac{2\times \zeta }{h_{\mathrm{PCC}}}\right))}{\frac{f}{2}+\frac{U_{m}\times P_{b}}{c_{1}\times d_{b}}}$$

where L, means CS [in]; f_t_ is the tensile strength of the concrete after 28 d (psi); C is the Bradbury’s curling/warping stress coefficient; σ_0_ is the Westergaard’s nominal stress factor (psi); ζ is the depth to steel layer (in); h_PCC_ is the slab thickness (in); f is the base friction coefficient; U_m_ is the peak bond stress (psi); P_b_ is the reinforcement ratio; c_1_ is the first bond stress coefficient, and d_b_ is the reinforcing steel bar diameter (in). 

The crack pattern of a CRCP pavement forms in the early stages of its operation (1–2 years after construction) and is influenced mostly by environmental conditions and traffic [1,23,25,38] and this makes it possible to calculate the average CS without considering the incremental time steps. The derivation of the input coefficients of Bradbury’s curling/warping stress coefficient (C), Westergaard’s nominal stress factor σ_0_, base friction coefficient (f), peak bond stress (U_m_), and first bond stress coefficient (c_1_) can be found in [24].

CW must also be calculated for each increment and not at a specific time, as in the case with CS. The maximum longitudinal tensile stress in the concrete slab can be calculated at the depth of the steel layer, which essentially depends on the bond between the reinforcing steel and the surrounding concrete which is influenced by three elements: (1) steel reinforcement bars, (2) environmental effects, and (3) subbase friction.

The expression used in the MEPDG for CW at the depth of the steel determination is presented in Equation (8).

(8) $${\mathrm{CW}}_{i}=\mathrm{CC}\times L\times \left(\varepsilon_{\mathrm{shr},i}+\alpha_{\mathrm{PCC}}\times \Delta T_{\zeta ,m}-\frac{c_{2,i}\times f_{\sigma ,i}}{E_{\mathrm{PCC},i}}\right)\times 1000$$

where, CC is the calibration constant; ε_shr,i_ is the unrestrained concrete drying shrinkage (10^−6^ in/in); α_PCC_ is the PCC coefficient of thermal expansion (°F^−1^); and ∆T_ζ,m_ is the drop in PCC temperature from zero-stress temperature at the depth of the steel at time of CW prediction (°F); C2 is the second bond stress coefficient based on the concrete strength and CS by 0 °C at the steel depth and zero temperature differential; EPCC is the concrete elastic modulus; f_σ_ is the maximum longitudinal tensile stress in the concrete at the steel level; and ε_shr,i_ is the unrestrained concrete drying shrinkage at the depth of the steel. Based on Equation (10) the CW depends on total strain (from shrinkage and temperature drop) at the depth of the steel and restrained longitudinal deformation caused by stresses in the concrete. The second bond stress coefficient for the monthly increment, i, is based on the concrete strength and CS and is calculated as:

(9) $$c_{2,i}=0.7606+1772.5{\times (\varepsilon }_{\mathrm{tot}\text{-}\zeta ,i}{)-2\times 10}^{6}\times {\left(\varepsilon_{\mathrm{tot}\text{-}\zeta ,i}\right)}^{2}+\frac{{9\times 10}^{8}{\times (\varepsilon }_{\mathrm{tot}\text{-}\zeta ,i})+149486}{{500\times U}_{m}}+\frac{{3\times 10}^{9}{\times (\varepsilon }_{\mathrm{tot}\text{-}\zeta ,i}{)}^{2}-{5\times 10}^{6}{\times (\varepsilon }_{\mathrm{tot}\text{-}\zeta ,i})+2020.4}{L^{2}}$$

where ε_tot-ζ,i_ is the total strain at depth of steel (10^−6^ in/in) from shrinkage and temperature drop. A well-functioning load transfer is the essential element for a good and long-lasting performance of CRCP. The decisive and variable factor of the total load transfer in CRCP is the load transfer in the area of the crack, which is supported by load transfer through the reinforcement and the base. The efficiency of the load transfer is identified in three different stages: stage (I) occurs when the CW is smaller than 0.5 mm (LTE 100%), stage (II) occurs when CWs are between 0.6 mm and 2.5 mm, and stage (III) represents the contribution of the foundation in the load transfer. This third stage corresponds to a CW larger than 2.5 mm [39]. Crack shear capacity deterioration potential increases once the ratio of the CW to PCC thickness approaches a value of 0.0037 [40]. The loss of shear capacity, ∆S_i_, at the end of a time increment due all load applications, j, is determined by using the following equations:

$$\frac{{\mathrm{CW}}_{i}}{h_{\mathrm{PCC}}}\;\Delta s_{i}$$

<1 0

(10) $$<3.8\;\Delta s_{i}=\sum_{j}(\frac{0.005}{1+1\times {\left(\frac{{\mathrm{CW}}_{i}}{h_{\mathrm{PCC}}}\right)}^{-5.7}})\times (\frac{n_{\mathrm{ji}}}{10^{6}})\times (\frac{\tau_{\mathrm{ij}}}{\tau_{\mathrm{ref},i}}{)\times \mathrm{ESR}}_{i}$$

(11) $$>3.8\;\Delta s_{i}=\sum_{j}(0.004+\frac{0.068}{1+6\times {\left(\frac{{\mathrm{CW}}_{i}}{h_{\mathrm{PCC}}}-3\right)}^{-1.98}})\times (\frac{n_{\mathrm{ji}}}{10^{6}})\times (\frac{\tau_{\mathrm{ij}}}{\tau_{\mathrm{ref},i}}{)\times \mathrm{ESR}}_{i}$$

where CW_i_ is the CW for each increment i (mil); h_PCC_ is the thickness (in); n_ji_ is the number of efficient axle load applications for increment i and load level j; τ_ij_ is the corner shear stress due to load j for month i (psi); τ_ref,i_ is the reference shear stress (psi); and ESR_i_ is the equivalent shear ratio for lateral traffic wander.

The load transfer factor efficiency due to aggregates interlock can be evaluated depending on transverse crack stiffness (J_c,i_).

(12) $${\mathrm{LTE}}_{c,i}=\frac{100}{{1+10}^{\left(\frac{0.214-0.183\times \frac{a}{l_{i}}-\log\left(J_{c,i}\right)}{1.18}\right)}}$$

The change in transverse crack width and wear-out of aggregate interlock contributed to the development of LTE due to aggregate interlock. For a prediction of damage to the total LTE due to aggregates interlock, the steel reinforcement and base layer for each monthly increment, i, during the design life should be calculated:

(13) $${\mathrm{LTE}}_{\mathrm{TOT},i}=100\times (1-\left(1-\frac{1}{{1+10}^{\left(\frac{0.214-0.183\times \frac{a}{l_{i}}-\log\left(J_{c,i}\right)-R}{1.18}\right)}}\right)\times \left(1-\frac{{\mathrm{LTE}}_{\mathrm{Base}}}{100}\right))$$

where a is the radius for loaded area (in) (the typical value for a loaded area is 6 inches); l_i_ is the radius of relative stiffness computed for the monthly increment i [in]; J_c,i_ is the transverse crack stiffness for the monthly increment i; R is the residual factor to account for residual load transfer provided by the steel reinforcement (=500P_b_ − 3); P_b_ is the longitudinal reinforcement (%); and LTE_Base_ is the LTE contributed by the base layer (%) (20% for granular base, 30% for ATB/CTB, and 40% for LCB). 

The aim is to keep LTE_TOT_ above 95% at all times to ensure adequate serviceability. After determining the LTE for each monthly increment, i, the traffic parameter for damage and shear loss computation should be settled. The fatigue damage caused by repeated loading of heavy axles influenced the development of punchouts. To predict the CRCP punchout, the accumulated fatigue damage from all previous monthly increments, i, is calculated:

(14) $${\mathrm{FD}}_{i}=\sum_{i}D_{i}$$

where FD_i_ is the accumulated fatigue damage at the end of the ith increment; and D_i_ is the damage for monthly increment, i, accumulated for all vehicle classes, j, as a relationship between the number of applied axle load applications of the jth magnitude evaluated during the ith traffic increment and the number of allowable axle load applications of the jth magnitude evaluated during the ith traffic increment to crack initiation in PCC. Then, the number of medium and high-severity punchouts based on the calibration database can be calculated [40]. A correlation function by using the predicted fatigue damage and the observed number of punchout was developed as follows:

(15) $${\mathrm{PO}}_{i}=\frac{A_{\mathrm{PO}}}{{1+\alpha }_{\mathrm{PO}}\cdot {\mathrm{FD}}_{\mathrm{PO}}^{\beta_{\mathrm{PO}}}}$$

where PO_i_ is the total predicted number of punchouts per mile at the end of ith monthly increment; FD_PO_ is the accumulated fatigue damage; and A_PO_, α_PO_, and β_PO_ are the calibration constants for punchout function of 195.789, 19.8947, and −0.526316, respectively. 

To cover the desired reliability, standard deviations must be added accordingly and punchouts at any given level of reliability are calculated based on the mean predicted value by using the expression:

(16) $${\mathrm{PO}}_{R,i}{=\mathrm{PO}}_{i}{+z}_{p}\times (-0.00609{\times \mathrm{PO}}_{i}{}^{2}+0.58242{\times \mathrm{PO}}_{i}+3.36789)$$

where PO_R,i_ is the number of punchouts under the selected level of reliability (mi^−1^); and z_p_ is the standardized normal deviate corresponding to reliability level. 

Smoothness as an indicator of serviceability is essentially dependent on any change in the longitudinal profile over time and traffic due to the development of distresses and foundation movements:

(17) $${\mathrm{IRI}}_{i}{=\mathrm{IRI}}_{0}+3.15{\times \mathrm{PO}}_{i}+28.35\times \mathrm{SF}$$

(18) $$\mathrm{SF}=\mathrm{AGE}\cdot (1+0.556\cdot {\mathrm{FI})\times (1+P}_{200}{)\cdot 10}^{-6}$$

where IRI_i_ is the IRI at the end of the monthly increment *i* (in/mi); IRI_0_ is the initial IRI (in/mi); SF is the site factor; AGE is the pavement age (years); FI is the freezing index (°F days); and P_200_ is the percent of subgrade material passing through a No. 200 sieve.

## 4. Evaluation of CRCP Sections

In the following section, the evaluation of sections based on the introduced design methods of the AASHTO 86/93 and the MEPDG is carried out to compare both methods regarding different material features, climate, and traffic load on the considered European CRCP sections and to examine the performance of test tracks and, if necessary, to create improvement alternatives. The predicted CW and CS were calculated according to both design methods and compared with real values recorded on site.

### 4.1. AASHTO 86/93

The minimum and maximum values for the CS and maximum values for the CW and SS have to be inserted into the AASHTO method and the minimum and maximum percentage of reinforcement are determined. In the following, according to AASHTO 86/93, a range between 1.07 and 2.44 m (3.5 and 8 ft) was assumed for the CS, a maximum value of 1 mm (0.04 in) for CW, and a maximum value of 407 MPa (59 ksi) to 455 MPa (66 ksi) for SS, depending on the tracks. The minimum required amount of steel is selected as the largest among the three criteria and the maximum allowable amount of steel is based solely on a minimum range of selected CS. Table 2 summarizes the required range of reinforcement ratio according to AASHTO 86/93 for the investigated routes.

With a high amount of heavy traffic load on the A5 near Darmstadt, the maximum number of equivalent 10 t axle loads corresponds to a short design life, which is not sufficient for a motorway. The design of such a motorway requires a level of reliability of 95%, which corresponds to a correction of 1.645 standard deviations. Furthermore, the slab thickness of 24 cm (9.45 in) is not sufficient because the American design, for example, recommends a slab thickness of at least 28.6 cm (11.25 in) for a design of over 100 million equivalent single axle loads. The design mean cylinder compressive strength of 38 N/mm^2^ (C30/37) chosen is lower than, for example, E313, which has a mean cylinder compressive strength of 53 N/mm^2^ (C45/55). To consider the design parameter, a design with a slab thickness of 28.6 cm (11.25 in), a compressive strength of 43 N/mm^2^ (6.237 psi), and a reliability of 50% would achieve 173.6 million equivalent 10t axle loads, which would correspond to a design life of 45.6 years. Due to an inaccurately installed CTB on A5 Darmstadt, the concrete slab is partly thicker than intended (by 2–3 cm on average), which caused the reinforcement ratio to drop to 0.67%. This means that the reinforcement is no longer centered in relation to the concrete cross-section. Even if all criteria are fulfilled, it shows the strong influence of execution on the success of the CRCP design. The crack pattern on site also shows short CS, indicating low reinforcement ratio. Using Equation (2) a reinforcement ratio of 0.67% would result in a crack spacing of 139.6 cm (4.58 ft), but the measured crack spacing in subsection II was only 50.9 cm (1.67 ft). For the design of the reinforcement in subsection III (CRCP + geotextile + CTB) and IV (CRCP + CTB), which differ from the first two only in the use of a CTB instead of ATB, similar conditions arise as for the first two subsections, since only a minimally lower wheel load results from the higher modulus of subgrade reaction, as the CTB is less sensitive to temperature than the ATB. The target range of reinforcement is between 0.57% and 0.77%, which is covered by the design (0.75%). Incidentally, the CS values for these two subsections were similarly low as for subsection II (CRCP + geotextile + AB). Subsection V (CRCP + geotextile + BC) with a lower modulus of subgrade reaction shares the weak results comparing with other subsections on A5 Darmstadt, as only stabilized soil was used as the base layer and therefore it performs weakest with a maximum number of equivalent 10 t axle loads of 7.0 million. The limits for the steel design in subsection V (CRCP + geotextile + BC) were determined to be between 0.56 and 0.75%, so the reinforcement design lies exactly on the limit of a too-short CS, which also occurred in subsections II to IV.

For the motorway A4, the number of permitted equivalent 10t axle loads dropped to 10 million. The AADTT at the time of construction in 2005 was 5700 trucks per day with a growth factor of 2% per year. It can be seen that the actual executed design of 0.76% meets the CW and allowable SS requirements because the required range of longitudinal reinforcement would be designed according to AASHTO with a ratio between 0.70 and 0.92%.

Due to low traffic volume on the access road Geseke, selected compressive strength of 43 MPa and the slab thickness of 22 cm (8.66 in), a maximum equivalent 10t axle load of 29.7 million can be achieved. In the first 12 years since commissioning, the road was subjected to a total of 2.77 million equivalent 10 t axle loads, i.e., less than 10% of the total number of possible axle loads. The executed ratio of longitudinal reinforcement of 0.75% is more or less exactly in the middle of the computed range. The transverse reinforcement would be designed according to AASHTO with a ratio of 0.088% and spacing of 65.28 cm (25.7 in). The construction was executed with a ratio of 0.15% and a spacing of 59.94 cm (23.6 in). The crack surveying in 2019 showed a mean CS of 86.51 cm (34.06 in) and a mean CW of 0.41 mm (0.016 in) [21]. 

The permissible number of equivalent 10t axle load applications on E313 in Belgium is approximately 23.3 million. The section was commissioned in 2012 with 5750 trucks per day and lane at an annual growth rate of 2.2%. On E313, as test section, two different depths of notches (30 mm and 60 mm) at a distance of 120 cm were made as a crack initiation. The limits for the reinforcement design were determined to be between 0.82 and 1.06% (designed 0.74%), which is high because of the selected mean compressive strength of 53 N/mm^2^. The last condition survey in 2019 showed a mean crack distance of 118 cm (46.46 in) on this section. 

The evaluation for track A5 near Bruchsal was nearly the same as for the track near Darmstadt. Every subsection must be investigated separately in theory. However, subsections I (washed concrete + CRCP + geotextile + BC), III (SMA + CRCP + geotextile + BC), and IV (thin overlay + CRCP + geotextile + BC) can be merged (besides the overlay). Subsections II (SMA + CRCP + AB + BC) and V (grinding + CRCP + geotextile + BC) are investigated individually. For subsections I, III, and IV, the target range for the reinforcement ratio is between 0.54 and 0.74%, and for subsection II with AB the design range for the steel ratio is between 0.56 and 0.76%. Subsection V has a thicker slab of bound cement of 25.5 cm (10.04 in) and the reinforcement ratio is determined to be between 0.59 and 0.80%. 

According to the AASHTO 86/93, the required steel percentage on the Geseke should be between 0.66 and 0.87% (designed 0.82%). The calculated reinforcement ratio on A5 Darmstadt should be between 0.57 and 0.77%, on the A5 Bruchsal between 0.54 and 0.74%, on the Geseke between 0.66 and 0.87%, on the A4 between 0.70 and 0.92%, and on the E313 between 0.82 and 1.06%. These criteria were carried out on the A5 Bruchsal and A5 Darmstadt with 0.75%, on Geseke with 0.82%, on A4 with 0.76%, and on E313 with 0.74%.

### 4.2. MEPDG

The development of the punchout, CW, CS, and LTE models applied by the MEPDG to each variable were studied and some of the input parameters from the examined tracks are summarized in Table 3 to investigate the design method and performance regarding MEPDG.

**Figure 9:**
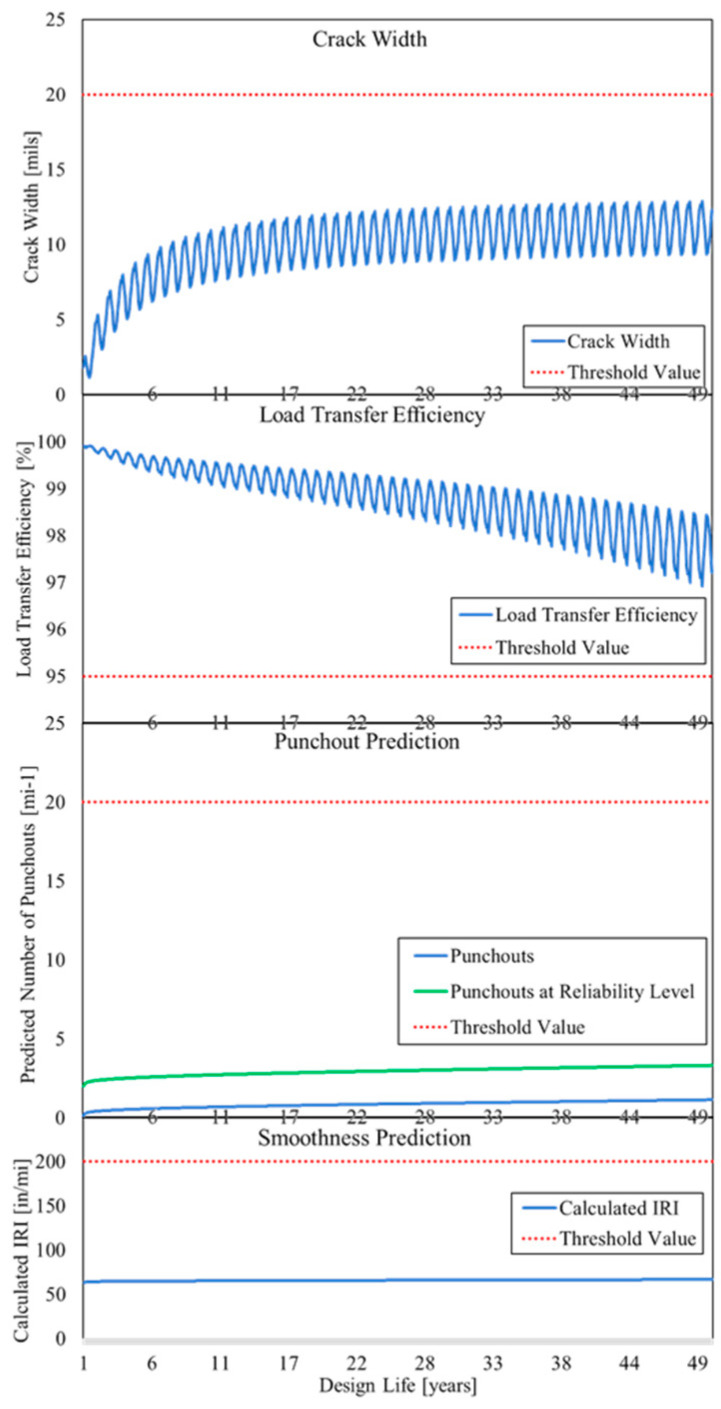
Variation of: CW, LTE, punchout prediction, and smoothness prediction over time from MEPDG on Geseke.

The performance predictions of five tracks and subsections with different input parameters were evaluated and analyzed. The results of the MEPDG design method display the IRI and punchout rates over time under consideration of 90% reliability. By extension of predicted IRI and punchout from specific limits at the end of the design life, the input parameters or selected variants must be adjusted. The development of the CW, LTE, punchout, and IRI of Geseke over 50 years of service life are shown in Figure 9.

The sections of Geseke performed very well over the 50 years of operation, far from the limits of serviceability. Nevertheless, it was also noticeable that with longer service life, the development of punchouts (the limit criterion for punchouts of national road is 20 punchouts per mile) accelerates, even if only slightly in this case. The values of CW and LTE are below the limit criterion during the design life. The seasonal course of the two values is recognizable, with a small CW in summer due to the expansion of the concrete due to the higher temperatures and consequently a high LTE, which is low in winter when the CWs are larger. The average CS of Geseke is 86.5 cm (34.6 in), which is just below the recommended minimum value of 107 cm (3.5 feet), therefore, the input data must be adjusted or new construction options must be examined.

**Figure 10:**
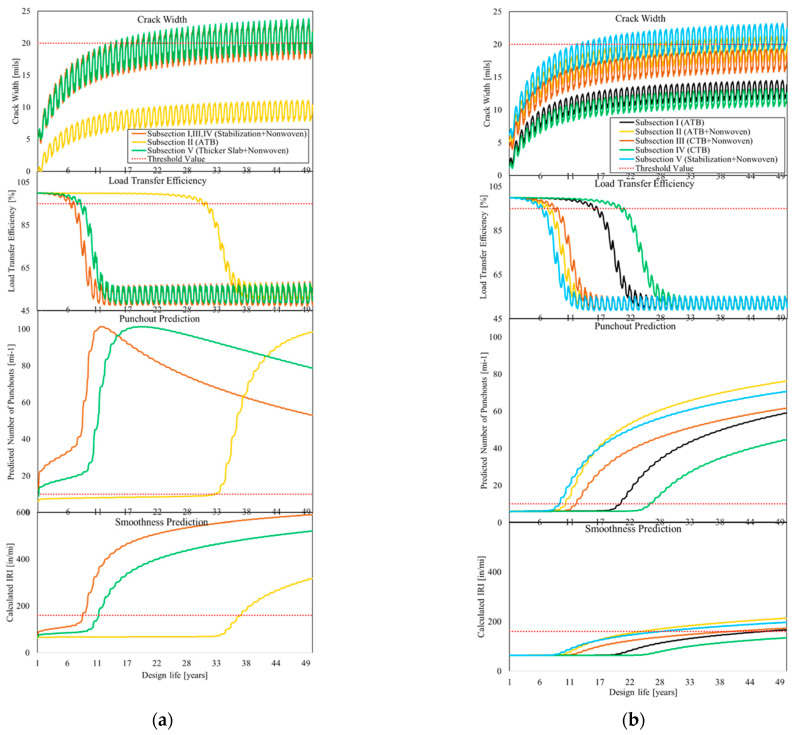
Variation of: CW, LTE, punchout prediction and smoothness prediction over time from MEPDG, (**a**): A5 Bruchsal, and (**b**): A5 Darmstadt.

The prediction of the punchouts and developing of CW, LTE, and IRI for the five subsections on A5 Darmstadt and A5 Bruchsal are shown in Figure 10.

On the A5 Bruchsal subsections I (washed concrete + CRCP + geotextile + BC), III (SMA + CRCP + geotextile + BC) and IV (thin overlay + CRCP + geotextile + BC), which can be merged (besides the overlay), subsections II (SMA + CRCP + AB + BC) and V (grinding + CRCP + geotextile + BC) are investigated individually and the massively better performance of the pavement structure without the geotextile (subsection II) is evident. Even considering the level of reliability, a design life of almost 35 years is achieved here, while the other subsections can in principle be regarded as unusable from the time of commissioning. Even the minimally thicker plate (1.5 cm (0.59 in) thicker) in subsection IV cannot compensate for this. Incidentally, the number of punchouts when taking the level of reliability into account goes down again after a certain value because the equation for the standard deviation (Equation (13)) has a zero at 101.11 punchouts. Since the number of punchouts cannot decrease without carrying out repair measures, the equation is no longer applicable at this value. However, the end of serviceability is reached much earlier. Considering CW development during the design life, the results transfer positive results. Subsection II (CRCP + AB + BC) gets maximum CWs of just over 10 mils even in winter. The other subsections reach the critical value of 20 mils in winter for the first time after about 12 years. The LTE of subsections with geotextile breaks down significantly just before the end of the design life after 10 years. 

**Figure 11:**
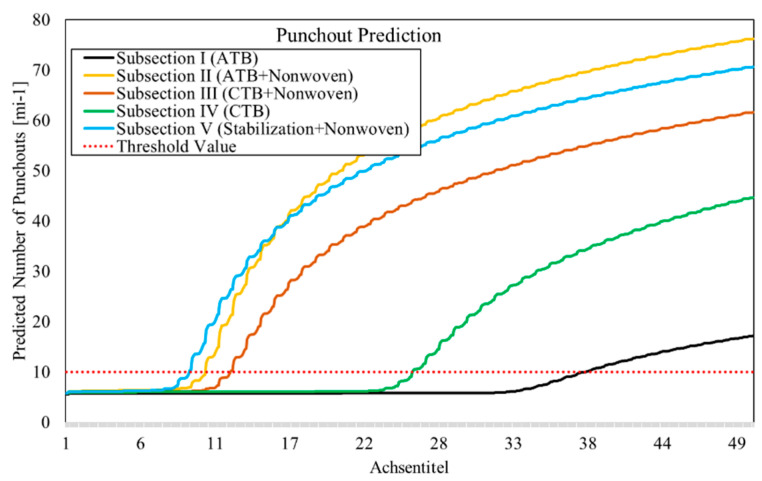
Prediction of punchout by increased slab thickness on the A5 Darmstadt.

On the A5 Darmstadt, the subsections without non-woven geotextile (subsection I and IV) performed significantly better, and secondly, the subsections with CTB (subsections III and IV), due to better friction, perform better than those with ATB (subsections I and II). The design life of the subsections is between 10 and 26 years depending on the limit criterion for 10 punchouts per mile on motorways. The reasons for short service life lie mainly in the slab thickness, which is below the AAHSTO recommendation and has a significant influence on the prediction result. Figure 11 shows the result of punchout prediction on the A5 Darmstadt by increasing the slab thickness.

For example, if the slab thickness is increased by 2.54 cm (1 in) to 26.54 cm (10.45 in) and the concrete cover of the reinforcement layer remains the same, the time until the threshold value is exceeded can be almost doubled.

The prediction of development of the IRI is directly dependent on the number of punchouts, however, with stretched design can live from 23 to over 50 years on this test track depending on subsections. The subsections with the geotextile quickly show a massive loss in load transfer, which ultimately has a negative effect on the development of the punchouts. Subsections I (CRCP + AB + BC) and IV (CRCP + CTB + BC) transfer the best results compared with the other subsections on the A5 Darmstadt. In terms of CW, on the other hand, subsections I and IV succeed in remaining permanently below the limit value, subsection III (CRCP + geotextile + CTB + BC) also remains below it, and subsection II (CRCP + geotextile + AB + BC) remains below the limit value in the summer months. However, according to the American code of practice, serviceability can only be ensured if the value of the CW remains permanently below the limit value, irrespective of the season. 

**Figure 12:**
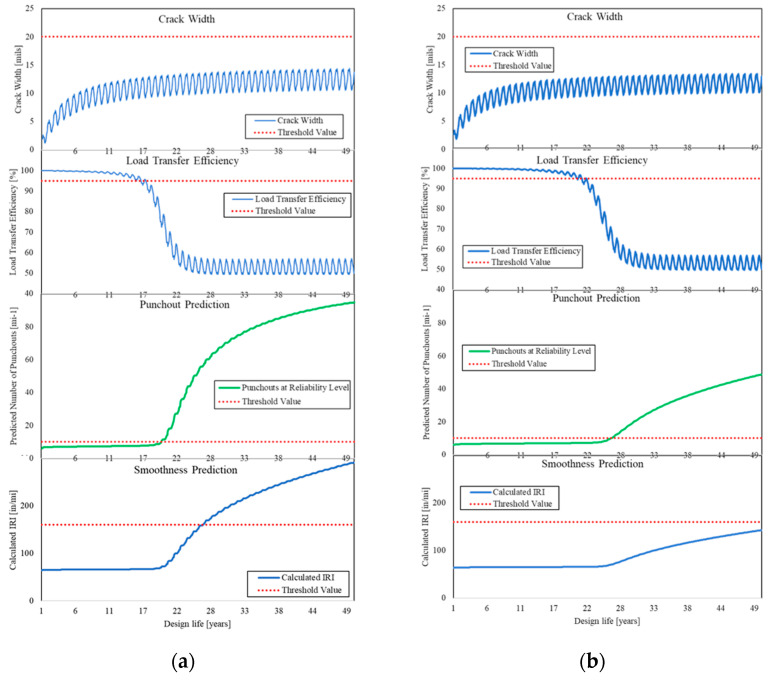
Variation of: CW, LTE, punchout prediction and smoothness prediction over time from MEPDG, (**a**): A4, and (**b**): E313.

The variation of the CW, LTE, punchout, and IRI of A4 and E313 over 50 years of service life are shown in Figure 12.

A comparison of the results between A4 and E313 consisting of CRCP on lean concrete makes the influence of 5 cm asphalt interlayer on E313 clear. On the A4, without any interlayer, CRCP and lean concrete are connected and operate as a single layer. Therefore, the degree of reinforcement reduces, as it is not used for the CRCP itself but for the CRCP and the underlying layer. MEPDG predicts for E313 a service life of 28 years with a reliability of 90% considering the punchout development although the limit value is exceeded after approximately 20 years at A4. Considering the prediction of smoothness, A4 hit the threshold value after almost 27 years although E313 has a service life of more than 50 years.

**Figure 13:**
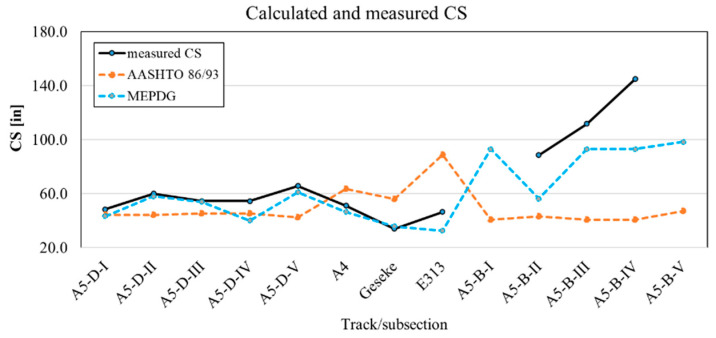

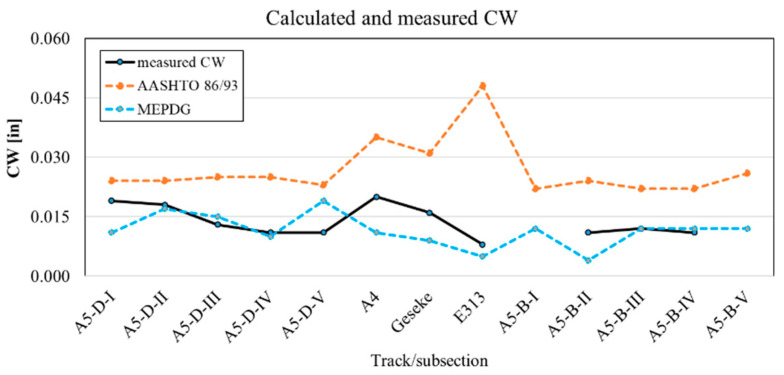
Calculated and measured CS and CW on examined sections.

Parallel to the determination of the punchout development and IRI on the considered routes, the CS and CW were estimated according to the AASHTO 86/93 and MEPDG and compared with measured CS and CW on the examined sites. Figure 13 shows the measured and calculated values for CS and CW.

Although there is a wide range of values, the MEPDG method seems to provide results that are closer to reality (measured values). The calculated CS on the A5 Darmstadt (MEPDG) is between 101.6 cm (40 in) and 155 cm (61 in). The results according to MEPDG are sometimes different from those according to AASHTO, but overall, they are closer to the measured values in all sections (except CS on the A5 Darmstadt section I and IV). The performance criteria are incrementally calculated for each month with the procedure according to MEPDG, the calculated values can also be output for comparison with the measured values for CW at the same time as the actually measured values as a function of the operating time. The calculated CS on the A5 Bruchsal for the subsections with geotextile are very high in the range of the maximum recommended CS of 243.8 cm (8 ft). Similarly, for the Geseke, the E313 also has an average CS below the recommended value of 106.7 cm (3.5 ft) and requires an adjustment in the design. The results of the CW of the MEPDG model seem to be more similar to the measured values on site.

## 5. Conclusions

Rigid pavements with CRCP are extremely durable when properly constructed, used, and maintained. The characteristic of CRCP (CS, CW, and crack LTE) is dependent on the performance prediction of punchout and IRI. The punchout, crack LTE, CW, and CS were evaluated based on two CRCP design procedure and the results were compared with the real CS and CW recorded on the test sections in three European countries with different construction details. The interactions between geometrics, material properties, traffic, and environmental conditions in the MEPDG method are more pronounced than in the AASHTO guide but both procedures are theoretically considered in the calculation of ε_tot−∆max_ for Westergaard’s factor. An increase in the amount of steel decreases the spacing between the cracks, leading to tighter CWs and more sustained load transfer, decreased IRI, and a lower number of punchouts. An increase in thickness can reduce the number of punchouts significantly and an increase in the depth of the steel from the slab surface results in an increase in punchouts and terminal IRI. The prediction values based on the MEPDG method follow the measures results more closely. Especially, the CS of the MEPDG model match more than the CW with measured values. The CS and CW based on MEPDG is significantly lower than the observed CS and CW on the test tracks. Although the AASHTO 86/93 is an imprecise approach which considers important variables such as environment or slab/base friction and influence of level of reliability to a certain limit and tends to underestimate the required steel, the MEPDG analyses vehicle type/load, material parameters and pavement characteristics, climate, and performance prediction. A strong reduction in friction between the slab and base by the geotextile is not recommended and the application of a hydraulically bound layer or asphalt layer under the concrete provides longer service life regarding punchout prediction. Beyond the type of base layer used, the compressive strength, subgrade reaction, and traffic load also have influence on the performance of the designed CRCP.

The prediction of CS and CW based on the MEPDG matched closely the measured data from the test sections. Despite positive experiences and developments, there are some deficiencies in the AASHTO, such as construction conditions and fatigue cracking from the bottom, which have important roles in the design of CRCP and are not taken into account in either procedure.

## Figures and Tables

**Table 1:** Technical data of the CRCP test tracks.

No	Tracks	Year	Length (km)	AADTT (Truck/24 h)	Total Load (m)	Cement	Reinforcement Ratio (%)		Slab Thickness (cm)	f_cm_ (MPa)
							Longitudinal	Transverse		
1	A5 Darmstadt	2004	1.52	7873	63	I	0.75	0.15	24	38
2	A4	2005	1.1	5700	45	I	0.76	0.07	23	40
3	Geseke	2009	1.1	450	2	I/II	0.82	0.15	22	43
4	E313	2012	7	5750	23	III	0.74	0.35	25	43/53
5	A5 Bruchsal	2015	2.27	10,060	29	I/III	0.75	0.14	24	38

**Table 2:** Design criteria and required range of steel percentage based on AASHTO 86/93.

	A5 Darmstadt		A5 Bruchsal		Geseke		A4		E313	
	Longitudinal Reinforcement (%)									
	Min	Max	Min	Max	Min	Max	Min	Max	Min	Max
CS (ft)	0.48	0.77	0.45	0.74	0.56	0.87	0.60	0.92	0.72	1.06
CW (in)	0.57	-	0.54	-	0.65	-	0.70	-	0.82	-
SS (ksi)	0.56	-	0.53	-	0.66	-	0.67	-	0.81	-
Range	0.57	0.77	0.54	0.74	0.66	0.87	0.70	0.92	0.82	1.06

**Table 3:** PCC mixture input parameter.

Parameter	Symbol	Units	Tracks				
			A5-D	A4	Geseke	E313	A5-B
PCC 28 day compressive strength	f′_c28_	psi	5076.3	8702.3	6236.6	6671.7	5076.3
PCC elastic modulus	E_PCC,i_	psi	4,319,423.2	4,728,230.3	4,787,689.3	4,951,868.3	4,319,423,2
PCC 28 day tensile strength	f_t,i_	psi	451.2	971.8	500.2	517.3	451.2
PCC modulus of rupture	MR_i_	psi	676.9	886.2	750.2	775.9	676.9
Ultimate shrinkage	ε_∞_	-	683.4	513.5	663.2	622.5	546.7
PCC temperature at set time (zero stress)	T_set_	°F	84.1	68.8	84.1	64.4	71.4
Bond slip coefficient	k_1_	psi	594,945.9	1,019,909.6	730,933.5	781,923.2	594,945.9
Peak bond stress	U_m_	psi	1189.9	2039.8	1461.9	1563.8	1189.9
Poisson’s ratio	µ_PCC_	-	0.2	0.2	0.2	0.2	0.2
LTE of base	LTE_Base_	%	granular base: 20%; ATB/CTB: 30%; LCB: 40%				
Base friction coefficient	F	-	ATB: 7.5; CTB: 8.9; soil cement: 7.9; LCB: 6.6				

## Data Availability

Not applicable.

## Abbreviation

Abbreviation	Explanation
CS	Crack spacing
CW	Crack width
SS	Steel stress
JPCP	Jointed plain concrete pavement
CRCP	Continuously reinforced concrete pavement
LTE	Load transfer efficiency
AASHTO	American Association of State Highway and Transportation Officials
MEPDG	Mechanistic Empirical Pavement Design Guide
AADTT	Average annual daily truck traffic
IRI	International roughness index
